# Culturally tailored interventions for ethnic minorities: A scoping review

**DOI:** 10.1002/nop2.733

**Published:** 2020-12-09

**Authors:** Jee Young Joo, Megan F. Liu

**Affiliations:** ^1^ Gachon University College of Nursing Incheon Korea; ^2^ School of Gerontology Health Management College of Nursing Taipei Medical University Taipei Taiwan

**Keywords:** culturally competency care, culturally tailored intervention, culturally tailored research, ethnic minority, health disparity, scoping review

## Abstract

**Aim:**

This scoping review identifies strengths and weakness of culturally tailored interventions for ethnic minorities’ care in the United States. It reviews recently published studies to improve understanding of these interventions for future research and practice.

**Design:**

Scoping review.

**Methods:**

By searching five electronic databases—CINAHL, PubMed, Ovid, Scopus and Web of Science, this review located 58 empirical studies published between 2015–2019. This review was guided by the PRISMA statements.

**Results:**

The review identified four weaknesses and five strengths of culturally tailored interventions. Weaknesses included unclear guidelines, low attention and retention rates, failure to measure processes and inadequate training for healthcare providers. The intervention strengths were culturally respectful and patient‐centred care, healthy lifestyle promotion, increased family and community supports, technology use for efficient and timely care and increased knowledge of disease by participants.

## 1 INTRODUCTION

In the United States, diverse ethnic minority groups are growing fast. In 2045, it is projected that 49.3% of the US population will be composed of Hispanic or Latinx, African American, Asian and multiracial populations (Frey, 2018), up from 41.8% in 2019 (United States Census Bureau, 2019). These populations are often underserved by healthcare systems because of economic and language barriers, meaning they frequently experience low‐quality care (Torres‐Ruiz et al. 2018). One consequence of the combination of increasing minority populations and persistently bad care is that the number of high‐risk patients—patients who typically experience high individual healthcare costs and economic losses (Joo & Liu, 2020)—in minority populations is increasing. One promising way to improve healthcare equity and quality for minority populations is by employing culturally tailored interventions (Torres‐Ruiz et al. 2018). Culturally tailored interventions have been shown promising care coordination that improving access to healthcare systems and clinical outcomes to ethnic minorities (Joo, 2014; Joo & Liu, 2020; Torres‐Ruiz et al. 2018).

### 1.1 Background

Cultural tailoring of interventions means “the adaptation of the study design, materials and other components of the intervention to reflect cultural needs and preferences at the population level” (Torres‐Ruiz et al. 2018, p. 3). The term *cultural tailoring* is used interchangeably with *cultural competency*, *culturally appropriate* and *cultural targeting* (Joo, 2014). Since the Healthy People 2030 goal for improving health and well‐being in the United States demonstrated the importance of patient‐centred research (HealthyPeople.gov, 2020), the need for cultural tailoring of interventions, studies and practices for populations with ethnic minorities has increased (Torres‐Ruiz et al. 2018).

Despite growing ethnic minority populations and widespread adoption of culturally tailored interventions in the United States, little is known about how effectively and efficiently culturally tailored interventions have been conducted or how the interventions have influenced overall healthcare delivery to ethnic minorities. While some systematic reviews of culturally tailored interventions exist, no review has yet given a broad overview of these interventions for ethnic minorities in the United States. This scoping review conducts that broad overview to identify the interventions’ weaknesses and strengths as a care coordination strategy and to present recommendations for future research, practices and healthcare policy.

## 2 METHODS

### 2.1 Aim

This scoping review aims to identify strengths and weakness of culturally tailored studies for ethnic minorities in the United States and to make recommendations for future research and practice. To provide recent available evidence, this review targeted studies of culturally tailored interventions conducted in the United States within the last 5 years, between January 2015–April 2020.

### 2.2 Study design

The study design employed a scoping review methodology and was guided by Arksey and O’Malley's methodological framework for a scoping review (2005). A scoping review collates and synthesizes broad areas of a particular topic of research to identify gaps in current studies and inform future research (Arksey & O’Malley, 2005; Cacchione, 2016; Levac et al., 2010; Nam et al., 2015). In addition, this review was guided by the Preferred Reporting Items for Systematic Reviews and Meta‐Analyses (PRISMA) statement (Page et al. 2020).

This review followed the five‐step process for scoping reviews recommended by Arksey and O’Malley (2005) and Levac et al. (2010): (1) identify the research question; (2) identify relevant studies; (3) select relevant studies; (4) chart the data; and (5) collate, summarize and report results.

#### 2.2.1 Identify the research questions

The research question of this scoping review was “What issues have culturally tailored interventions presented to ethnic minority care in the United States in the last 5 years?” In this study, *issues* refer to intervention strengths or weaknesses, implementation gaps and process and outcome‐measure difficulties. This review had two specific corollary research questions: “What are the implementation weaknesses of these culturally tailored interventions?” and “What strengths do these interventions share as a care coordination?”

#### 2.2.2 Identify and select relevant studies

To comprehensively identify studies in recent literature, this review conducted search strategies in three steps. First, English peer‐reviewed journals were searched using the electronic databases CINAHL, PubMed, Ovid, Scopus and Web of Science. Search terms using Medical Subject Headings (MeSH) and keywords were used with Boolean operators and appropriate truncations: (culturally tailored intervention) OR (cultural competency) OR (culturally targeting) OR (culturally appropriate) AND (ethnic monitories) OR (immigrants). The following additional keywords were also used for searching: (implementation) OR (strategy) OR (vulnerable population) OR (cultural humility) OR (cultural congruence). The search included descriptive studies; empirical studies, including experimental studies; qualitative studies; and mixed methods and secondary analysis studies. All studies were published between January 2015–April 2020 and were conducted in the United States. The search was conducted initially in April 2020 by the first author and a nursing researcher (professor emeritus), and a confirmation search was redone in May 2020.

Second, this review used purposive sampling to add any existing literature that might identify issues of culturally tailored intervention to ethnic minorities and gaps in culturally tailored intervention research. This step included citation tracking of the relevant literature, including the studies identified in step 1. Reviews, editorial comments and policy papers were excluded. Supplementary Table 1 summarizes the inclusion and exclusion criteria of this scoping review.

**Table 1:** Themes in selected texts

Main theme	Subtheme	Relevant study quotation
Weaknesses	Unclear guidelines	“Based on the literature, there are no clear published guidelines to develop culturally appropriate dietary interventions among minority populations” (Aycinena et al, 2017, p. 19) “We suggest developing a protocol for standardized interventions that includes regular training of staff members, through which intervention quality could be maintained despite varying competencies among intervention staff” (Im et al. 2018, p. 432) “the integration of cultural competency standards into everyday practices in health care organizations remains challenging…. The National Standards for Culturally and Linguistically Appropriate Services (CLAS) were developed by the Department of Health and Human Services’ Office of Minority Health over a decade ago, and serve as a framework to assist hospitals in developing organizational cultural competency.” (Ogbolu et al., 2018, p. 4) “Expanded model exploration and theoretical framework testing to identify the most relevant constructs and pathways for transitioning knowledgeable African American young adults to improve behaviors are also recommended for future research studies” (Zellner et al. 2015, p. 9)
Low attention and retention rates		“Reasons stated for nonparticipation included lack of interest in dietary change, illness, and work constraints” (Bernard‐Davila, 2015, p. 2) “A lower dose of physical attendance was delivered than initially intended due to low attendance rates… one of the major barriers to attending the present intervention was transportation to the intervention site” (Burkart et al., 2017, p. 91) “Notable challenges included slow recruitment, difficulty identifying eligible children and low family participation in intervention activities” (Crespo et al. 2018, p. 707) “We had missing data for 17% (*n* = 11) of the sample who did not have subsequent follow‐up clinical encounters within the Denver health system” (Fischer et al., 2015, p. 665) “The trial had good retention at 6 months, but only 38% of participants attended all 9 sessions” (Greenlee et al. 2015, p. 720) “Most participants (Asian American cancer survivors) worked during the day; as a result, they requested coaching and support sessions in the evening, often outside the working hours of intervention staff. Despite the benefits of personalized coaching and support, such a problem could result in participant burden, which raised questions about the sustainability of the intervention” (Im et al. 2018, p. 431) “Mean attendance in the intervention group was 40%: 11 women attended no group sessions, 12 women attended 1 to 5 sessions, and 9 women attended 6 sessions or more” (Joshi et al. 2018, p. 3) “Attrition is also a concern…. While we had few challenges recruiting participants into the study, at one year follow up, we were able to obtain follow up questionnaires on 575 participants out of 745 (77%)” (Langford et al., 2015, p. 254) “Our average attendance was 7.4/12 or 61.6%, with a majority (55.5%) of the intervention group participants attending 9 to 12 sessions. Longer duration (12 sessions versus 6 sessions), as well as the timing of our program (December–February), may have impacted our attendance rate” (Patel et al., 2017, p. 6)
	Failure to measure processes	“more studies are needed which demonstrated how process evaluation may be used to understand implementation of childhood obesity programs, especially in underserved and ethnic minority populations” (Alia et al. 2015, p. 3) “maintaining fidelity at the group level was somewhat more challenging” (Alia et al. 2015, p. 16) “lack of direct assessment of how cultural adaptations may have impacted intervention effectiveness, lack of fidelity ratings for all CST (a culturally tailored pain coping skills training) sessions” (Allen et al. 2019, p. 11) “Although there is low quantity of culturally‐tailored mother‐daughter physical activity interventions, the reported mixed results could be due to the implementation of these interventions or other process evaluation issues (i.e., study fidelity)” (Burkart et al., 2017, p. 88) “there is limited data describing the process evaluation of African‐American parent‐child interventions” (Burkart et al., 2017, p. 89) “there may be a need for a research coordinator to be present in clinics to provide support and to ensure the fidelity of study design and administration.” (Felicitas‐Perkins et al. 2017, p. 176) “there is a relative paucity of scientifically rigorous evaluations of a culturally tailored, theoretically driven caregiver intervention with Latino populations” (Gonyea et al., 2016, p. 301) “Additionally, there was not an equal dose of CT (clinical trial) educational messages for the comparison churches, which limits internal validity” (Langford et al., 2015, p. 254)
	Inadequate training for healthcare providers	“assessing culture was a challenge and requires highly trained evaluators. Advanced training related to identifying and describing cultural topics/issues may be important for future studies” (Alia et al. 2015, p. 16) “Participants (RNs) expressed a desire to provide culturally competent care and recognized the benefits of including cultural considerations in their patients’ care and the nurses’ own needs for more education on how to provide culturally respectful nursing care” (Coleman & Angosta, 2017, p. 685) “The need for cultural considerations in the nursing process needs to be addressed in academic and staff development curricula” (Coleman & Angosta, 2017, p. 687) “Another practical issue was difficulties in recruiting, training, and retaining competent staff for the intervention” (Im et al. 2018, p. 432) “The chief nurse executives raised concerns that the current training in cultural competency was insufficient, not cost effective, and that providers needed opportunities for safe and practical learning” (Ogbolu et al., 2018, p. 5)
Strengths	Culturally respectful and patient‐centred care	“Effective communication between nurses and patients and families with limited English proficiency is essential to provide safe, culturally competent and patient‐centered care” (Coleman & Angosta, 2017, p. 679) “Offering the intervention in Spanish removed linguistic barriers and allowed participants to better articulate the nature of their caregiving experience within their own cultural framework and addressed a critical need ‘to be heard.’ Further, the importance of personalismo was incorporated into all stages of the research project” (Gonyea et al., 2016, p. 298) “The community health worker intervention was designed to address these norms related to physical activity and diet in a culturally relevant manner” (Islam et al. 2018, p. 108) “All education modules are designed to be relevant and person‐centered” (Lynch et al. 2016, p. 6) “In order to achieve a more culturally‐appropriate approach in our study, all of our research project leaders and interventionists underwent training, prior to the start of the trial, for culturally responsive care and building trust and strengthening provider/patient relationships, and hopefully increase the study participants’ adherence to the interventions (Nguyen‐Huynha et al. 2019, p. 89) “We designed an individualized diet and lifestyle telephone coaching program that provided ongoing support and helped to identify barriers specific to blacks with hypertension, focused on culturally appropriate education materials” (Nguyen‐Huynha et al. 2019, p. 91) “Most of the chief nurse executives surveyed expressed a sincere desire to be educated prior to a patient's admission (if feasible given the circumstance), to better prepare for that particular patients’ cultural needs” (Ogbolu et al., 2018, p. 7) “Findings indicate that program involvement bolsters parents’ cultural pride and their efforts to share Latino values and traditions with their teens and increases teens’ involvement in cultural practices” (Sieving et al. 2017, p. 761) “The a Depression Education Fotonovela (a culturally appropriate depression education intervention) differs from typical patient education materials by incorporating surface and deep‐level cultural elements including the use of simple language, attractive visuals, cultural norms, and educational messages that target specific misconceptions and attitudes about depression and depression treatment common among Hispanics” (Sanchez et al. 2017, p. 3)
	Healthy lifestyle promotion	“Each participant's new awareness of the ability to take control of his or her disease emerged as participants engaged in blood glucose self‐monitoring at home and began feeling empowered and motivated to make lifestyle changes” (Brunk et al. 2017, p. 190) “Overall, mothers expressed satisfaction with the intervention program and the instruction provided during the dance class. Components of most importance to mothers included homework help for their daughters, a fun culturally‐tailored program, a convenient location, and increased time spent with their daughter…” (Burkart et al., 2017, p. 91) “The findings support the necessity of a culturally tailored Internet Cancer Support Groups for Asian American breast cancer survivors to reduce their support care needs, physical and psychological symptoms, and uncertainty, subsequently enhancing their quality of life” (Chee et al., 2016, p. 626) “positive trends were noted in the intervention group in physical activity, diabetes self‐management (general diet and self‐checking of feet), family support and the physical component of health‐related quality of life” (Hu et al. 2016, p. 307) “Women in the (culturally tailored) intervention group ate more daily servings of fruits and vegetables than women in the control group, and most importantly, they ate more dark‐green and deep‐yellow vegetables” (Greenlee et al. 2015, p. 719) “participants (South Asians) expressed enthusiasm about activities teaching them how to incorporate physical activity in daily life…. Participants reported that the intervention had a positive impact on their overall quality of life and well‐being” (Jayaprakash et al. 2016, p. 809) “all (100%) reported they would recommend the culturally‐relevant Facebook and text‐message delivered physical activity program to a friend” (Joseph et al., 2015, p. 12) “Culturally‐tailoring intervention activities have the potential to increase the acceptability and uptake of a behavioral interventions, which can ultimately lead to positive changes in the targeted and behavioral outcome of interest” (Joseph et al., 2015, p. 16) “the DM (diabetes)‐related quality of life score was significantly improved, and, more importantly, these statistically significant improvements were sustained for 12 months” (Kim et al. (2015, p. 735)
	Increased family and community supports	“it is important to understand regional cultural differences and the needs of the community through preliminary research” (Burkart et al., 2017, p. 91) “Additionally, the importance of social support during a quit attempt was discussed, and the counselor worked closely with the participant to identify ways to enhance social support during a quit attempt, particularly through family support” (de Dios et al. 2019, p. 6) “Active and Healthy Families (a culturally tailored, family‐based program) participants and providers reported that promotoras (community health workers) bridged cultural, linguistic, and other divides between the health care system and participating families” (Falbe et al. 2017, p. 735) “Importantly, the study was guided by a coalition of community stakeholders…” (Islam et al. 2018, p. 108) “Our partnership illustrates a promising approach for addressing disparities in adolescent sexual health outcomes, with researchers and community partners working as equal partners to address issues of relevance to the community” (Sieving et al. 2017, p. 761) “Participants noted that physical and social environment of the church fostered relationships between church members and provided social support for health promotion” (Whitney et al. 2017, p. 4) “patient engagement (through focus groups) and community partnerships (with a faith‐based organization) facilitated the translation of an effective health care–based intervention into a real‐world community setting” (Whitney et al. 2017, p. 6)
	Technology use for efficient and timely care	“Perhaps through the tailored SMS messages, they increased their competence and levels of autonomous motivation to continue their Mexican Americans, as they all maintained BP control and high medication possession ratios” (Chandler et al. 2019, p. 10) “As this study reported, Internet Cancer Support Groups could be acceptable by racial/ethnic minority populations, and could be effective in enhancing their survivorship experience” (Chee et al., 2016, p. 626) “results of this study show promise in the utilization of a culturally‐tailored DVD to improve clinical trial participation among Filipino cancer patients” (Felicitas‐Perkins et al. 2017, p. 177) “For example, an at‐home instructional DVD was created in Bengali and shared with treatment group participants to encourage physical activity that was both culturally accessible and low in cost” (Islam et al. 2018, p. 108) “technology‐based platforms can provide researchers the ability to reach a large number of people at a relatively low cost” (Joseph et al., 2015, p. 2) “All materials will be available in multiple languages on an informational website developed to be culturally tailored for community members, parents, families and caregivers” (Karasz & Bonuck, 2018, p. 6) “The utility of advanced communication technology in health care is in facilitating medical encounters, increasing access to health care services, and broadening availability of resources, even among underserved populations” (Lynch et al. 2016, p. 2)
	Increased knowledge of disease by participants	“Using bilingual/bicultural interventionists and teaching strategies targeted at low literacy participants with diabetes experienced a 187% increase in average diabetes knowledge score from baseline to post‐intervention and a 157% increase from baseline to 1‐month follow‐up” (Hu et al. 2016, p. 308) “Study results show that the hands‐on skills and knowledge building approach to dietary change was effective in this patient population, who had low levels of health literacy and acculturation” (Greenlee et al. 2015, p. 719) “Significant intervention effects were shown for type 2 diabetes knowledge (knowledge scale and knowing what HbA1c is)” (Islam et al. 2018, p. 106) “After the intervention, participants in the intervention group had significantly higher knowledge of Filipino adolescent behavioral health disparities and higher perceived susceptibility to adolescent risky sexual activity and illegal drug use” (Javier et al. 2019, p. 1) “SHIP‐DM (a community‐based, multimodal behavioral self‐help intervention program) also improved DM‐related psychobehavioral outcomes, including self‐efficacy of DM self‐management and DM(diabetes) knowledge” (Kim et al. (2015, p. 735) “The intervention's cultural tailoring helped participants feel more comfortable and understood by providing relevant Filipino educational materials and coaching from Filipino staff” (Maglalang et al. 2017, p. 150) “Our study provides evidence that exposing Hispanic women to culturally tailored programs aimed at improving HIV knowledge, when combined with strategies that address skills, can significantly reduce their participation in risky behaviors, reducing their risk of HIV infection” (Montano et al. 2019, p. 572) “Participants (Hispanic adults) in the classes showed increased knowledge on the material that was taught and reported behavior changes consistent with the strategies emphasized in the workshops” (Otilingam et al. 2015, p. 184)

**Figure 1:**
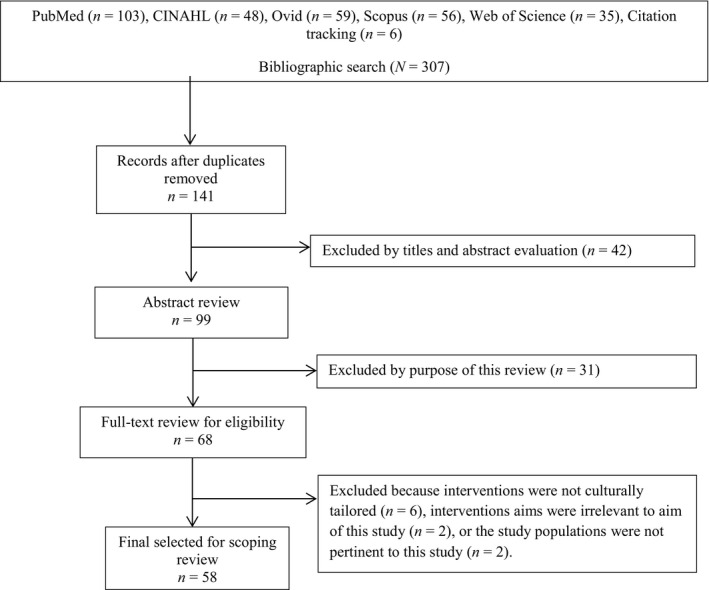
Flow chart of scoping review search process

Third, the first author and the aforementioned researcher selected the final studies for this scoping review. Initially, 307 references were identified from the five electronic databases, and after duplicates were removed, citations were tracked. The first author reviewed titles and abstracts of the studies and excluded by purpose of this review. Based on this review process, 68 studies were identified for full‐text review. After full‐text review, 10 studies were excluded for the following reasons: interventions were not culturally tailored, study aims were irrelevant to the aim of this study, or the study populations were not pertinent to this study. Ultimately, 58 studies were included in this scoping review (Figure 1). The first author and the nursing researcher reviewed the selection process once again, and agreement was made. Because this study was a scoping review which aims to map the existing literature, quality assessment of included studies was not conducted (Arksey & O’Malley, 2005; Nam et al., 2015).

#### 2.2.3 Chart, collate and summarize results

A table was developed to chart, collate and summarize results from the 58 included studies. The tabulation included the following standardized information: year of publication, author(s), sample, study design, purpose and major findings. The first author and the second author then independently examined the table for accuracy and to identify for common themes (Levac et al., 2010; Nam et al., 2015). Key ideas were summarized into subthemes that were then grouped into two main themes: strengths and weaknesses of culturally tailored interventions. A summary of major themes and subthemes is provided in Table 1. The first author and the second author cross‐checked the themes against the table to ensure accuracy and trustworthiness.

## 3 RESULTS

Table 2 summarizes characteristics of the 58 studies included in this review; Supplementary Table 2 presents details of the studies. All the studies were conducted by a multidisciplinary team including researchers from disciplines such as nursing, medicine or social work. Analysis of the studies identified four weaknesses and five strengths of culturally tailored interventions (Table 1).

**Table 2:** Characteristics of studies included in the scoping review

Category	Detail	Total
Study design	Experimental (include RCTs)	28
	Qualitative (mixed method)	10
	Descriptive (protocol explanation studies, descriptive analysis, etc.)	15
	Pilot	4
	Secondary analysis	1
Study sample	Chronic illnesses^a^	25
	High‐risk patients (e.g. HIV, STI)^a^	18
	Cancer survivors	10
	Nurses or healthcare practitioners^a^	6
	Others (caregivers, research team members)	3
Target ethnic minority	African Americans^a^	20
	Hispanic or Latinx^a^	26
	Asian Americans or Asian immigrants^a^	14

Note

HIV = human immunodeficiency virus, RCT = randomized controlled trial, STI = sexually transmitted infection.

^a^ duplicated

### 3.1 Study characteristics

The studies in this review included were primary and empirical: quantitative studies, quasi‐experimental studies, RCTs, qualitative studies, mixed‐method studies, descriptive studies, pilot studies and secondary analyses were all represented in the selection. Targeted ethnic minorities included African Americans; Hispanic or Latinx peoples, including immigrants; and Asians, including immigrants. Participants in the studies were diagnosed with chronic illnesses, high‐risk patients, cancer survivors, nurses or healthcare practitioners and caregivers (see Table 2).

### 3.2 Weaknesses of culturally tailored interventions

Four weaknesses of culturally tailored interventions were identified: (1) unclear guidelines; (2) low attention and retention rates; (3) failure to measure processes; and (4) poor cultural competency training.

#### 3.2.1 Unclear guidelines

Several studies reported that guidelines for conducting culturally tailored interventions were lacking. Indeed, no studies in this review followed process recommendations or guidelines for standard care. Ogbolu et al. (2018) pointed out that it is difficult to apply the standard guidelines to everyday nursing practices, especially in acute clinical care. Aycinena et al. (2017) noted that clear published guidelines for culturally competent care, especially dietary interventions for ethnic food and norms, were lacking. Im et al. (2018) observed that standardized protocols of culturally tailored interventions are needed to ensure intervention quality.

#### 3.2.2 Low attention and retention rates

Many studies reported participants’ low attendance and retention rates in culturally tailored interventions. Several of the experimental studies in review reported high attrition rates for their participants. Moreover, low retention rates applied to African Americans, Hispanic or Latinx participants and Asians. Burkart et al. (2017) and Crespo et al. (2018) reported low attendance rates as one of the major barriers to conducting their culturally tailored interventions. Retaining participants for the entirety of an intervention is imperative to ensure research is valid and reliable and to gain adequate power of sample (Langford et al., 2015). However, as intervention durations increased, retaining participants became more difficult (Greenlee et al. 2015; Patel et al., 2017). For example, Greenlee et al. (2015) reported high attendance rates at the beginning of the study that by the end dropped to 38%.

Studies gave several reasons for low attendance and high attrition rates of participants. Most participants came from underserved populations with low incomes and had limited access to transportation, inhibiting their ability to attend intervention sites (Burkart et al., 2017; Im et al. 2018). Bernard‐Davila et al, 2015) and Burkart et al. (2017) demonstrated that sustained interventions created a burden on participants who had to work for their living.

#### 3.2.3 Failure to measure processes

In this review, many studies reported lack of intervention process measurement as a barrier of culturally tailored interventions. Measuring intervention dosage and fidelity to evidence‐based practice is important, but none of the included studies applied intervention dosage analysis, maintained fidelity to interventions or made process evaluations using specific measurement tools.

Among experimental studies in this review, Alia et al. (2015) and Allen et al. (2019) demonstrated that the most challenging part of conducting culturally tailored interventions was maintaining fidelity. Langford et al. (2015) reported that they could not conduct the study with equal dose of usual care to the control group and observed that this threatened the study's internal validity.

Only a few studies measured intervention processes. For example, Burkart et al. (2017) evaluated quantity and fidelity of intervention. As a result of this analysis, they found that low intervention dosage affected their outcome. Alia et al. (2015) evaluated intervention processes on their trial implementation (Alia et al. 2015).

Other studies stressed the importance of developing and validating evaluation tools for culturally tailored interventions (Allen et al. 2019; Gonyea et al., 2016). Felicitas‐Perkins et al. (2017) recommended that reporting intervention fidelity was important to ensure experiments are conducted as planned.

#### 3.2.4 Inadequate cultural competency training

Several studies reported difficulty training healthcare providers and intervention staff to conduct culturally tailored interventions. Although some studies hired bilingual healthcare providers to communicate and provide culturally competent care, most did not.

Concerns about culturally competent healthcare providers were reported. Coleman and Angosta (2017), a qualitative study, reported that nurses wanted to provide culturally respectful care, but the healthcare system gave them insufficient training to do so. Ogbolu et al. (2018) said that cost and time limited their ability to solve issues with culturally competent care. Nurses were educated either at an orientation session or annually and had few chances to receive continuing cultural competency training (Ogbolu et al., 2018). Other studies reported that recruiting and training staffs to deliver or observe culturally competent interventions was challenging (Alia et al. 2015; Im et al. 2018).

### 3.3 Strengths of culturally tailored interventions

Five strengths of culturally tailored interventions were identified: (1) culturally respectful and patient‐centred care; (2) healthy lifestyle promotion; (3) increased family and community supports; (4) technology use for efficient and timely care; and (5) increased knowledge of disease.

#### 3.3.1 Culturally respectful and patient‐centred care

In the included studies, interventions were designed and delivered relevant to ethnic minorities’ cultural mores (Islam et al. 2018; Nguyen‐Huynha et al. 2019). Sanchez et al. (2017) incorporated cultural factors such as language, cultural framework, including culturally appropriate educational materials for their Hispanic or Latinx patients with depression. Sieving et al. (2017) similarly considered patients’ cultural values and traditions when designing their practices.

The culturally tailored interventions were also patient‐centred. Studies assessed ethnic minorities’ critical needs and delivered care based on those needs (Gonyea et al., 2016; Lynch et al. 2016; Nguyen‐Huynha et al. 2019). Ongoing supports and barrier assessments for participants also enabled patient‐centred care (Nguyen‐Huynha et al. 2019).

#### 3.3.2 Healthy lifestyle promotion

The culturally tailored interventions under review were applied to ethnic minority groups with a variety of high‐risk factors and illnesses. Many studies reported that culturally competent care was effective in promoting healthy lifestyles (Brunk et al. 2017; Burkart et al., 2017; Chee et al., 2016; Hu et al., 2016).

In Jayaprakash et al. (2016), a qualitative study, South Asian participants said that the intervention positively affected their lifestyle, including their physical activity and improved their well‐being. Similarly, Kim et al. (2015) reported significantly higher quality of life scores in the culturally tailored intervention group (Korean Americans with T2DM) than in the comparison group; the higher scores were sustained for 12 months. In Joseph et al. (2015), African American participants were very satisfied with how culturally tailored care affected their physical activity and they said they would recommend the programme to others. Brunk et al. (2017) reported that participants felt empowered to control their glucose level and motivated to change their lifestyles because of the intervention. Finally, in Hu et al. (2016), Hispanic or Latinx participants with T2DM changed their diets and glucose monitoring through intervention and thereby increased their health‐related lifestyle changes.

#### 3.3.3 Increased family and community support

The reviewed studies demonstrated the importance of involving families and building coalitions with communities in the interest of improving efficient care (de Dios et al. 2019; Islam et al. 2018). De Dios et al. (2019), for example, stressed social and family supports because they are connected with effective care of ethnic minorities. With these family members’ supports, ethnic minority participants obtained pertinent care and did not drop out of the study (de Dios et al. 2019). In populations from cultures that place significant importance on family values, such as Hispanic or Latinx and Asian communities, family helps deliver culturally based care effectively (Falbe et al. 2017; Islam et al. 2018).

Studies also emphasized the value of community supports. For example, one study that targeted African Americans with T2DM connected with churches (Whitney et al., 2017) to better facilitate effective health care. Falbe et al. (2017) designed their study to provide community referrals and reduce healthcare gaps between different settings. Sieving et al. (2017), a pilot study, argued that involving community partners could reduce structural healthcare barriers, suggesting culturally tailored care could be a promising approach to reduce gaps in the healthcare system.

#### 3.3.4 Technology use for efficient and timely care

Several studies delivered culturally accessible information using technological tools such as mobile text messages, DVDs and websites (Chandler et al. 2019; Chee et al. 2016; Islam et al. 2018). Chee et al. (2016) showed a website for ethnic minority cancer groups was effective in increasing support for cancer survivors. Islam et al. (2018) used bilingual technology platforms to encourage participants to change their diets and to exercise. Karasz and Bonuck (2018) made a website that was available in multiple languages and that included culturally tailored information to help ethnic minority patients stay healthy.

Studies found that technological tools could provide efficient and timely care to ethnic minorities (Chandler et al. 2019; Felicitas‐Perkins et al. 2017) at relatively low cost (Joseph et al., 2015). Since many ethnic minorities in the United States are underserved by the healthcare system, technological solutions could increase their overall access to healthcare services (Lynch et al. 2016).

#### 3.3.5 Increased knowledge of disease

The last frequently reported strength of culturally tailored care was increased knowledge of diseases and health care. Many of the interventions included health care or health improvement education programmes with easy‐to‐understand bilingual and bicultural educational materials (Hu et al. 2016; Islam et al. 2018; Javier et al. 2019; Kim et al. 2015). Through these programmes, intervention participants had significantly greater knowledge about their risks of disease compared with other groups or with themselves before the intervention (Montano et al. 2019; Otilingam et al. 2015). Hu et al. (2016), aiming to increase diabetes knowledge among low‐literacy Hispanic patients diagnosed with T2DM, showed a 157% knowledge increase after one month in the intervention group. Greenlee et al. (2015) demonstrated that a knowledge‐building approach was effective in increasing knowledge and self‐management skills among participants with limited health literacy. Likewise, Filipino participants in Maglalang et al. (2017) said that they felt comfortable and knowledgeable with culturally appropriate educational materials.

## 4 DISCUSSION

This scoping review highlighted several weaknesses and strengths of culturally tailoring interventions to ethnic minorities (Table 1). The review's findings lead to several implications for research, practice and health policy.

### 4.1 Implications for research

First, culturally tailored intervention need clearer intervention guidelines. Although strategies for culturally tailoring interventions have been proposed (Kreuter and Wray, 2003) and while standards for culturally and linguistically competent services have been developed (Health & Human Services: Office of Minority Health, 2018), many studies reported a lack of intervention protocol as a barrier to culturally tailored intervention research. Ogbolu et al. (2018) said the standards were difficult to apply in acute healthcare settings and no other included practical research studies followed these standards. More practical, refined and pragmatic guidelines are required. Thus, further research to develop standards for culturally tailored interventions that can be applied in a variety of healthcare settings with multiple ethnic minorities is needed. To assist in this project, cultural tailoring strategies also need better models or theoretical frameworks. Most of the studies in this review adopted theories from other disciplines. As one study emphasized, exploring theoretical models and testing them are important for culturally tailored intervention research (Zellner et al. 2015).

Second, culturally tailored interventions need better process measuring tools. Intervention researchers should explicitly describe the details of intervention dosage and how they achieve equal doses to participants (Sidani et al., 2010). Increasing the evidence of intervention dosage and connecting that evidence to patients’ outcomes can improve the overall evidence base of research and ensure high quality of health care (Sidani et al., 2010). However, studies in this review reported that lack of process measuring was a barrier (Alia et al. 2015; Allen et al. 2019; Gonyea et al., 2016; Huang & Garcia, 2018; Joo & Liu, 2020). Developing reliable and validated tools to measure culturally tailored interventions is therefore recommended.

Third, to ensure sample power culturally tailored interventions should find ways to keep participants from dropping out of studies and reduce attrition rates (Gray et al., 2016). Studies reported that high attrition and low retention rates were challenges of culturally tailored intervention research. High participant attrition is understandable: participants may have transportation restrictions that limit their access to intervention sites or have low incomes that require them to work for their living. Research with these populations needs to consider such constraints such as by having intervention sites that are accessible to ethnic minorities or by providing transportation help. Further research into how culturally tailored intervention studies might be efficient and valuable to ethnic minorities is also recommended.

Finally, the researchers should conduct further research into how best to use technology for intervention delivery. Several studies used mobile phone text messages for follow‐up services and websites to provide culturally and linguistically tailored education. Applying those technologies could prove to be efficient and cost‐effective care strategies (Islam et al. 2018; Joseph et al., 2015). Research should explore such applications of technology and connect them to patients’ outcomes for advanced and innovative culturally tailored interventions.

### 4.2 Implications for nursing practice

From this review come two recommendations for culturally tailored nursing practices. First, nurses need better education and training to practice culturally sensitive nursing. In addition to the findings from this review, previous studies have reported that nurses had insufficient education and training in their bachelor's curriculum (Debesay et al., 2014; Hart & Mareno, 2014). To ensure patient‐centred practice, as part of their continuing education nurses and other healthcare practitioners should have chances to regularly train in culturally sensitive care for ethnic minorities.

Second, practical guidelines for healthcare practitioners should be better standardized. In this scoping review, most interventions were delivered by multiple healthcare providers, including nurses, community health workers, social workers and physicians. Studies indicated that the lack of practical guidelines were obstacles for healthcare providers (Coleman & Angosta, 2017; Ogbolu et al., 2018). Similarly, several studies reported that a lack of clinical practical guidelines for culturally competent care (Joo & Liu, 2020; Marion et al. 2016). Clear, evidence‐based practical manuals are needed to improve the practices of nurses and other healthcare providers.

### 4.3 Implications for health policy

Culturally competent care should be supported by healthcare policymakers at all levels of government. First, research into culturally competent interventions should be funded. Studies reported that culturally tailored intervention studies require sustained support and funding (Chee et al. 2016; Kwon et al. 2015); however, few of the studies in this review were funded federally—most were funded by short‐term grants. More and more long‐term funding opportunities are needed to demonstrate the effectiveness of culturally tailored interventions for ethnic minorities.

Hospitals and healthcare practitioners should also be reimbursed for providing culturally tailored care. In the United States, there are financial penalties from the Center for Medicare and Medicaid Services if hospitals fail to provide patient‐centred, culturally appropriate care to patients (Ogbolu et al., 2018). Even with these penalties, nurse leaders in hospitals said it was difficult to provide culturally competent care in everyday practices (Ogbolu et al., 2018). Reimbursing providers of culturally tailored practices would increase the satisfaction of healthcare providers and improve quality of care for ethnic minorities.

### 4.4 Limitations of the review

There are three key limitations to this scoping review. First, this review was limited to studies that were conducted in the United States and only published in English. Second, the review did not include any government or policy papers, books or dissertations about culturally tailored interventions. Finally, in focusing on culturally tailored interventions in the care of ethnic minorities, the review did not isolate results by age group or by specific diseases. While the review included chronic illnesses and high‐risk populations, it could not include all diseases nor all ethnic minorities living in the United States.

## 5 CONCLUSION

This is the first scoping review to identify weaknesses and strengths of recent interventions culturally tailored to ethnic minorities in the United States. The review analysed 5 years’ worth of empirical studies and identified several intervention weaknesses that could pose risks for future ethnic minorities’ care delivery. Avoiding these risks requires rigorous research to provide the evidence base so culturally competent care can be applied. Strengths of culturally tailored interventions identified by this review can be used to advance care practice for ethnic minorities. Overall, this review finds that culturally tailored interventions have tremendous potential to reduce health disparities and improve quality of care for ethnic minorities. Broadly adopting these care strategies can help healthcare providers meet the goals of Healthy People 2030.

## 6 DATA AVAILABILITY STATEMENT/DATA ACCESSIBILITY STATEMENT

None.

## 7 DECLARATION OF CONFLICTS OF INTEREST

None.

## AUTHOR CONTRIBUTIONS

JYJ: Study design. JYJ, MFL: Data collection, analysis and synthesis. JYJ: Manuscript writing. JYJ, MFL: Critical revisions for important intellectual content. JYJ, MFL: Study supervision.

## ETHICAL APPROVAL

Because no human subjects were engaged in this study, no Institutional Review Board approval was required.

## Supporting information

Table S1‐2
