# Supplementary material for: Culturally tailored interventions for ethnic minorities: A scoping review
Source: Nurs Open. 2020 Dec 9;8(5):2078–90. doi: 10.1002/nop2.733 (PMC8363345; doi:10.1002/nop2.733)
Supplement: Supplementary file 1 — Table S1‐2 [file NOP2-8-2078-s001.docx]

Supplement Table 1. Inclusion and exclusion criteria

| Inclusion criteria | Exclusion criteria |
| --- | --- |
| Studies focused on interventions culturally tailored to ethnic minorities living in the United States | Studies conducted outside the United States |
| Empirical studies, including qualitative, quantitative, and mixed-methods studies or secondary analysis | Editorials, commentaries, or policy papers |
| Studies with culturally tailored interventions, development processes, or protocols | Studies that do not describe culturally tailored interventions or do not include ethnic minorities in the United States |
| Conducted in the United States | Studies in languages other than English |
| Studies targeting ethnic minorities living in the United States or healthcare providers caring for ethnic minorities | Reviews that include studies from outside of the United States |
| Studies published in peer-reviewed journals | Books or government documents |
| Studies published from January 2015 to January 2020 |  |

Supplementary Table 2. Details of selected culturally tailored intervention studies

| Study | Sample | Design | Purpose | Major findings |
| --- | --- | --- | --- | --- |
| Akintobi et al. (2016) | African American heterosexual males or females with HIV | Quasi-experimental | To examine the effectiveness of Color It Real (culturally tailored intervention designed to provide HIV education and address behavioral motivations) in increasing condom use and decreasing risky sexual behaviors | IG had significant increases in HIV transmission knowledge (*F* = 4.84, *p* = .0305) and intention to use condoms (*F* = 4.38, *p* = .0385). |
| Allen et al. (2019) | African Americans with knee or hip osteoarthritis | RCT | To examine a culturally tailored pain-coping-skills training program compared to a waitlist control group | The program did not significantly reduce pain severity but did improve key measures of pain coping and perceived ability to manage pain among participants. |
| Alia et al. (2015) | African American adolescents | Descriptive | To demonstrate how process evaluation is used to assess implementation of, and to provide formative feedback for, a culturally tailored, motivational plus family-based weight loss program | Results identify areas for program improvement related to delivery of multitheoretical and cultural tailoring elements. |
| Amirehsani et al. (2019) | Hispanic or Latinx patients with T2DM and their family members | Qualitative | To conduct focus groups with patients and their family members who participated in a culturally tailored, family-based diabetes intervention about their action plans for a healthier lifestyle | Participants created action plans that promoted healthier lifestyle behaviors individually and as a family. Six themes emerged from the data: healthier eating habits, increasing physical activity, taking care of my sugar, coping with emotions, for the family, and empowerment and increased self-efficacy. |
| Aycinena et al. (2017) | Hispanic or Latinx breast cancer survivors | Descriptive | To describe the use of the Nutrition Education DESIGN Procedure, a theory-driven approach, to inform the development of a culturally based dietary change program, ¡Cocinar Para Su Salud! (Cook for Your Health) | A systematically planned, evidence-based, culturally tailored dietary intervention was designed. |
| Bernard-Davila et al. (2016) | Urban Hispanic or Latinx breast cancer survivors | Descriptive | To understand factors related to recruitment to culturally based dietary modification trial | Reasons stated for nonparticipation included lack of interest in dietary change, illness, and work constraints. Compared to women who enrolled, nonenrollers were more likely to state that research is costly to participants (*p* = 0.03) |
| Briant et al. (2016) | Hispanic or Latinx patients with colorectal cancer in Washington State | Quasi-experimental | To assess changes in colorectal cancer awareness, knowledge and colorectal cancer screening-related behaviors, following the implementation of culturally tailored home health intervention | A culturally tailored colorectal cancer education facilitated by promotors (community health providers) in a rural environment is an effective way to increase colorectal cancer screening awareness, knowledge, and screening among participants. A statistically significant increase in colorectal cancer screening awareness and knowledge was observed. |
| Brunk et al. (2017) | Hispanic or Latinx patients with T2DM and low health literacy | Qualitative | To assess the feasibility of adapting a patient-centered culturally tailored educational intervention for T2DM self-management | A culturally tailored educational intervention promoting self-management of T2DM to participants revealed four themes: information and knowledge, motivation and barriers to change, experiences with new behaviors, and personal responsibility. |
| Burkart et al. (2017) | African American mother-daughter dyads | RCT | To describe process evaluation data including intervention fidelity, dosage, quality, participant responsiveness, and program reach for the Mothers And dauGhters daNcing togEther Trial (MAGNET) | Participants in the IG enjoyed participating in MAGNET > 90% of the time. Mothers (92%) indicated that they wanted to continue dance as a form of physical activity. Mothers expressed that transportation, time commitment, and assessments were barriers to participation. |
| Chandler et al. (2019) | Hispanic or Latinx adults with uncontrolled HTN and poor medication regimen | RCT | To conduct a 9-month culturally tailored mHealth medication regimen self-management program addressing BP control | At the 1, 3, 6, and 9-month time points, systolic BP averages were significantly lower in the IG than the CG (month 1: 125.3 vs. 140.6; month 3: 120.4 vs. 137.5, month 6: 121.2 vs. 145.7 mmHg; month 9: 121.8 vs. 145.7, respectively; all *p* < 0.01). Average medical regimen adherence, the IG ranged from 89.1 to 95.2% across the 9-month trial. The program was statistically and clinically significant reductions in systolic BP among participants. |
| Chee et al. (2016) | Asian American breast cancer survivors | RCT | To determine the preliminary efficacy of a culturally tailored registered nurse–moderated Internet Cancer Support Groups program in enhancing participants’ breast-cancer-survivorship experience | All users and experts positively evaluated the program and provided their suggestions for display, educational content, and user-friendly structure. Controlling for background and disease factors, the IG showed significantly greater improvements than the CG in physical and psychological symptoms and quality of life (*p* < 0.10). |
| Coleman & Angosta (2017) | RNs | Qualitative | To explore the lived experiences of acute-care bedside nurses caring for patients and their families with limited English proficiency when providing culturally competent care | Four themes emerged: nurses desired to communicate, connect, provide care, and provide cultural respect and understanding. |
| Crespo et al. (2018) | Overweight Hispanic or Latinx youth | RCT | To test the efficacy of a clinic-based intervention to lower BMI and improve body composition | There were no significant intervention effects on child BMI (*p* > 0.05); however, the IG children showed significantly (*p* < 0.05) lower total and trunk fat percentage compared with the CG. |
| de Dios et al. (2019) | Hispanic or Latinx smokers | RCT | To conduct a feasibility pilot randomized trial testing culturally tailored smoking cessation and adherence enhancement intervention | IG showed higher days of nicotine patch use than CG. At the 3-month follow-up, IG showed approximately 50% of the IG were smoking abstinent. |
| Desrosiers et al. (2019) | African American men who have sex with men | Pilot | To test whether a culturally tailored counseling center impacts participant access and uptake of HIV pre-exposure prophylaxis | At the end of the 3-month study, six participants in the IG compared with none in the CG had initiated HIV pre-exposure prophylaxis (*p* = 0.02). |
| DiClemete et al. (2015) | African American adolescent females (aged 14–18 years) | RCT | To examine an innovative culturally tailored, computer-delivered media-based strategy to promote HPV vaccine uptake for African American adolescent females | Participants in the IG were more compliant to vaccination relative to the CG (26 doses versus 17 doses; *p* = 0.12). Although not statistically significant, greater uptake of HPV vaccine was observed in the IG relative to the CG. |
| Falbe et al. (2015) | Hispanic or Latinx parent-child dyads | RCT | To test the impact of a family-centered, culturally tailored obesity intervention delivered through group medical appointments on BMI and other measures of cardiovascular risk | After 10 weeks of intervention, child BMI (kg/m2) decreased (−0.50) in the IG and increased (+0.32) in the CG. Children assigned to the IG also exhibited relative improvements over controls in BMI and triglycerides, but no significant between-group differences were observed for blood pressure or other fasting blood measures. |
| Falbe et al. (2017) | Low-income, overweight, and obese Hispanic or Latinx children aged 5–12 years and their families | Qualitative | To understand the Active and Healthy Families (a culturally tailored, family-based program) for addressing obesity disparities | Three main categories encompassing five themes emerged: (a) bridging communication by promotoras (community health workers); (b) promotoras’ personal qualities, including themes of kindness and caring and shared experiences with patients; and (c) impactful task performance, including themes of motivation, positive environment, and self-efficacy. |
| Felicitas-Perkins et al. (2017) | Filipino cancer patients | Pilot | To determine the effect of a culturally tailored educational DVD | A multilingual educational DVD to supplement clinical trial education may positively influence Filipino cancer patients to move forward with the decision to join a cancer clinical trial. However, health literacy may serve as a major barrier to actual enrollment into the particular clinical trial available to a patient. |
| Fischer et al (2015) | Hispanic or Latinx adults with serious illness | RCT | To determine the feasibility of a culturally tailored patient navigator intervention to improve palliative care outcomes | The intervention was found to be feasible and suggests improved palliative care outcomes for Hispanic adults facing advanced medical illness, justifying a fully powered RCT. |
| Greenlee et al. (2015) | Hispanic or Latinx breast cancer survivors | RCT | To examine the effect of a culturally based approach to dietary change on increasing fruit/vegetable intake and decreasing fat intake | At month 6, the IG compared to CG reported an increase in mean servings of fruits/ vegetables from baseline. The intervention was effective at increasing short-term fruit/vegetable intake. |
| Gonyea et al (2016) | Hispanic or Latinx Alzheimer’s caregivers | RCT | To test the effectiveness of Circulo de Cuidado, a culturally sensitive, cognitive behavioral group intervention, in supporting participants’ ability to manage neuropsychiatric symptoms of Alzheimer’s and to improve participant well-being | Compared with the CG, the IG reported lower neuropsychiatric symptoms in their relative, less caregiver distress about neuropsychiatric symptoms, a greater sense of caregiver self-efficacy, and less depressive symptoms over time. The study findings suggest the intervention has positive psychological benefits for Hispanic caregivers. |
| Hall et al. (2016) | African Americans | Descriptive | To address development of a culturally tailored, community-based intervention to raise awareness of brain health and increase engagement in behaviors to promote brain and overall health | Exposing vulnerable audiences to tailored and culturally appropriate brain health information may change peoples’ behavioral intent and ultimately health-protective behaviors. |
| Hu et al. (2016) | Hispanic or Latinx patients with T2DM and their family members | Quasi-experimental | To test efficacy of a family-based, culturally tailored intervention | IG improved in diabetes knowledge and diabetes self-efficacy over time (but did not sustain at 6-month follow-up). HbA1c was lower at 1-month follow-up. Family members had improvements in diabetes knowledge and physical health-related quality of life. |
| Im et al. (2018) | Research team members conducting a study among Asian American survivors of breast cancer | Qualitative | To identify practical issues in implementing a culturally tailored technology-based intervention | The study identified practical issues related to (1) technology literacy and preferences; (2) language issues; (3) cultural attitudes, beliefs, and values; (4) intervention staff competence; (5) security and confidentiality issues; and (6) time and geographical constraints. |
| Im et al. (2019) | Asian American breast cancer survivors | Randomized pretest/posttest group | To test the efficacy of a theory-driven culturally tailored intervention program on menopausal symptoms of participants | The IG showed a significant decrease in the distress scores of menopausal symptoms over time. Theory-based variables including attitudes, social influences and self-efficacy partially mediated the impact of the culturally tailored intervention on the distress scores of menopausal symptoms (*p* < 0.10). |
| Islam et al. (2019) | Bangladeshi immigrants with T2DM in New York City | RCT | To test the efficacy of a community-health-worker-led patient-centered lifestyle intervention on T2DM management | The average decrease in HbA1c was 0.2% greater for the IG than for the CG. |
| Javier et al. (2019) | Filipino caregivers of children ages 6–12 years | RCT | To test (1) the effectiveness of a theory-based, culturally tailored video versus a usual-care mainstream video on enrollment in an evidence-based parenting program and (2) the theoretical mediators of intervention effect | Postintervention, the IG had significantly higher knowledge of Filipino adolescent behavioral health disparities and higher perceived susceptibility to adolescent risky sexual activity and illegal drug use. |
| Jayaprakash et al. (2016) | South Asian immigrants and study staff | Qualitative | To understand a perception about the feasibility and efficacy of a community-based culturally tailored lifestyle intervention | Participants said that culturally tailored experiential activities helped increase knowledge and behavior change. Staff identified the need to reduce participant burden due to multicomponent intervention and agreed that the community-based organization needed greater financial resources to address participant barriers. |
| Joseph et al. (2015) | African American women | RCT | To evaluate a multicomponent, culturally relevant intervention designed to promote physical activity | A culturally relevant Facebook- and text message–delivered physical activity program was associated with several positive outcomes, including decreased sedentary behavior, increased light- and moderate-lifestyle intensity physical activity, enhanced psychosocial outcomes, and high participant satisfaction. |
| Joshi et al. (2018) | African American women who are overweight or with obesity | RCT | To test the feasibility of a culturally tailored adaptation of the Diabetes Prevention Program in an urban hospital setting | Women in the IG reported higher levels of satisfaction with the program, despite low attendance rates at group meetings. The intervention was not feasible because of these low rates of attendance and high rates of attrition after randomization. |
| Karasz & Bonuck (2018) | Low-income and at-risk mother-child dyads from South Asia | Descriptive | To address a protocol for reducing child obesity | A culturally tailored intervention will be applied to the at-risk immigrant South Asians designed to promote culturally competent, sustainable change. |
| Kim et al. (2015) | Korean Americans with T2DM | RCT | To test the effectiveness of a community-based, culturally tailored, multimodal behavioral intervention program | During the 12-month project, the IG demonstrated reductions in HbA1c. The differences between the IG and the CG were statistically significant. The IG showed statistically significant improvement in diabetes-related self-efficacy and quality of life when compared with the CG. |
| Kwon et al. (2015) | African American, Hispanic or Latinx, Chinese, and Korean seniors with HTN | Descriptive | To test a culturally tailored community-based walking intervention designed to reduce stroke risk by increasing physical activity | Trained case managers employ by the senior centers implement hour-long culturally tailored intervention sessions twice weekly for four consecutive weeks to the IG. Results from this trial will provide important insight into the design and effectiveness of sustainable community-based interventions aiming to reduce stroke risk and mitigate disparities among hypertensive ethnic minority seniors. |
| Langford et al. (2015) | African Americans | Quasi-experimental | To test the impact of a culturally tailored intervention aimed at increasing enrollment in a university-based clinical trials registry | Odds of verified enrollment were higher in the IG than the CG (OR = 2.95, 95% CI: 1.33–6.5, *p* = 0.01). Posttest self-reported enrollment in the registry was also higher among the IG than the CG (OR = 1.94, 95% CI: 1.08–3.47, *p* = 0.03). Willingness to participate in a future clinical trials was higher in the IG (β = 0.74, *p* = 0.02). |
| Larsen et al. (2015) | Hispanic or Latinx adults | RCT | To assess the costs and cost effectiveness of a Spanish-language print-based mail-delivered physical activity intervention | At six months, the intervention cost $29 per person per month, compared to $15 per person per month for wellness control. These costs fell to $17 and $9 at 12 months, respectively. While the intervention was more costly than the wellness control, costs per minute of increase in physical activities were lower in the intervention. |
| Lynch et al. (2016) | Low-income African Americans with poorly controlled T2DM | Descriptive | To address a protocol of culturally tailored diabetes education and skills training delivered via videoconferencing to improve health-risk behaviors and reduce cardiovascular disease risk outcomes | Culturally tailored components have evolved from understanding the food content, social practices, and acceptable forms of physical activity in southern urban African American culture. |
| Maglalang et al. (2017) | Filipino Americans with T2DM | Qualitative | To assess the acceptability and cultural relevance of the PilAm Go4Health program, a culturally adapted mobile health weight-loss lifestyle intervention that includes virtual social networking | Over half (*n* = 26, 57.8%) of the respondents found that a culturally tailored intervention program enhanced their engagement. A majority of the respondents (*n* = 29, 64.4%) expressed that they progressed from despair to self-efficacy as a result of their participation in the intervention. |
| McEwen et al. (2019) | Mexican American adults with T2DM and their family members | 2-group, experimental repeated-measures | To test the effects of a culturally tailored family-based self-management education and social support intervention on family social capital | The intervention demonstrated potential to improve social support for physical activity and family efficacy for diabetes management for participants. |
| Montano et al. (2019) | Hispanic or Latinx women | RCT | To evaluate the effectiveness of Salud, Educación, Prevención, y Autocuidad/Health, Education, Prevention and Selfcare (a culturally tailored intervention) to increase HIV/STI prevention behaviors delivered in a real-world setting | Significant outcome improvements were observed in the IG at 6 and 12 months’ follow-up. The adjusted prevalence of any condom use was 30% and 37% higher at each follow-up. The IG reduced HIV/STI risk-related behaviors. |
| Nguyen-Huynh et al. (2018) | Primary care physicians and their panels of HTN African American patients | Descriptive | To address a protocol of a culturally tailored diet and lifestyle-coaching intervention intended to improve BP control rates and to reduce disparities in HTN control | A culturally appropriate study intervention is a uniquely designed trial that included components from both pragmatic and explanatory methods. |
| Ogbolu et al. (2018) | Chief nurse executives | Qualitative | To examine participants’ perspectives on (1) the provision of culturally and linguistically appropriate services in hospitals and (2) barriers and facilitators associated with the implementation of culturally and linguistically appropriate services | Seven themes emerged: (1) lack of awareness of resources for health care organizations, (2) constrained cultural competency training, (3) suboptimal resources (cost and time), (4) mutual understanding, (5) limited workplace diversity, (6) community outreach programs, and (7) the management of unvoiced patient expectations. |
| Okoro et al. (2018) | African Americans with T2DM | Qualitative | To identify the key defining features of a culturally appropriate peer support program by exploring participants’ experiences related to assistance with daily disease management, emotional support, linkage to clinic care and community resources, and ongoing support | Three specific themes were identified: (1) healthy behaviors, (2) frequent telephonic contact, and (3) emotional support as a by-product of other support activities. |
| Otilingam et al. (2015) | Hispanic or Latinx women | RCT | To evaluate culturally tailored, theory-based nutrition education | There was statistically significant gain in health literacy, knowledge about dietary fat, and behaviors to reduce dietary fat in the IG compared with the CG. |
| Patel et al. (2017) | Asian Indians in an urban community | Quasi-experimental | To evaluate a community-based culturally appropriate lifestyle intervention program’s effectiveness at to reduce the risk for T2DM among participants | The IG lowered their HbA1c (*p* < 0.0005) and waist circumference (*p* = 0.04) significantly compared to the CG. |
| Pinsker et al. (2017) | Somali youth in Minneapolis, Minnesota | Descriptive | To develop a culturally targeted tobacco prevention intervention | Development of videos based on Somali culture and religion was facilitated through collaborating with trusted, existing community programs. |
| Printz (2019) | Asian American adolescents | Pilot | To examine the effect of a culturally tailored intervention aimed at encouraging vaccination for HPV | 76.3% of adolescents whose parents received the intervention went on to get at least 1 dose of the vaccine, compared with only 10% of adolescents whose parents did not receive the intervention. |
| Ramirez et al. (2019) | 300 Hispanic or Latinx breast, prostate, and colorectal cancer survivors | Descriptive | To address a protocol testing the efficacy of combining patient navigation-facilitated interventions | Culturally competent interventions using patient navigations have potential to address these needs and significantly improve Hispanic cancer survivorship. |
| Ravenell et al. (2015) | African American and Hispanic or Latinx patients | Descriptive | To test how effectively culturally tailored stroke education films change behavioral intent to call 911 for suspected stroke, compared to usual care | A culturally tailored stroke education film is highly scalable and easy to disseminate. |
| Sanchez et al. (2017) | Adult Hispanic or Latinx patients with depression | Descriptive | To test the effectiveness of a culturally appropriate depression education intervention to reduce stigma and increase uptake in depression treatment | This study protocol represents the first RCT of culturally adapted depression education among Hispanics in a primary care setting. |
| Schoenthaler et al. (2015) | Hispanic or Latinx patients with uncontrolled hypertension who are non-adherent to their antihypertensive medications | Descriptive | To test the effectiveness of a culturally tailored, practice-based intervention compared to usual care on medication adherence | If successful, findings from this study will provide salient information on the translation of culturally tailored, evidence-based interventions targeted at medication adherence and blood pressure control into practice-based settings for this high-risk population. |
| Shi et al. (2018) | Hispanic or Latinx breast cancer survivors | Secondary analysis | To examine psychosocial mediators of the effect of a culturally tailored dietary intervention on dietary change | At 12 months, the IG was associated with an increase in 0.5 servings per day fruit/vegetable intake through improved taste/snack preference for fruit/vegetable at 6 and 12 months (95%; CIs: 0.1–1.3, 0.0–1.4, respectively). |
| Shrestha et al. (2017) | Low-income African American older adults with worry/anxiety | Descriptive | To outline a study designed to test the effectiveness of a culturally sensitive intervention | The intervention will offer valuable information to help expand the reach of anxiety treatment among minority seniors living in underserved neighborhoods. |
| Sieving et al. (2017) | Hispanic or Latinx adolescents and their families | Pilot | To develop, implement, and test the feasibility and acceptability of a culturally tailored teen–parent health-promotion program | Compared to the CG, the IG reported substantially more involvement in activities celebrating Hispanic culture, and greater communication with their parents about sexual health topics. Pilot study findings confirm program feasibility and acceptability. |
| Weber et al. (2016) | Overweight or obese Asian Indian adults | RCT | To test the effectiveness of guideline-based, stepwise diabetes prevention by comparing the incidence of diabetes between the CG and the IG participants and by determining whether a culturally tailored lifestyle education curriculum intervention effects differ across baseline prediabetes type, HbA1c level, age, sex, BMI level, or family history of diabetes | A stepwise diabetes prevention program reduced the 3-year diabetes risk by 32% (95%; CI: 7–50) in participants. |
| Whitney et al. (2017) | African Americans churchgoers with T2DM | Qualitative | To describe the cultural tailoring of a clinic-based diabetes education program and to pilot the program | Participants described how religious beliefs play an active role in many aspects of diabetes care, including self-management behaviors, coping strategies, and patient/provider communication. |
| Yeh et al. (2016) | Chinese immigrants with prediabetes in New York City | RCT | To evaluate the effectiveness and feasibility of implementing a linguistically and culturally tailored diabetes prevention program | There was a significantly greater percent weight loss in the IG (−3.5 versus −0.1%; *p* = 0.0001) at 6 months, which was largely maintained at 12 months (−3.3 versus 0.3%; *p* = 0.0003). |
| Zellner et al. (2015) | African American urban young adults aged 18–24 years | Quasi-experimental | To test an age-specific and culturally tailored intervention designed to provide substance abuse and HIV education and reduce perceived stress | For the IG, perceived stress levels were significantly reduced by the end of the intervention (*p* = 0.020), condom use at last sexual encounter significantly increased (*F* = 4.43, *p* = 0.0360), and the IG participants were significantly less likely to drink five or more alcoholic drinks in one sitting (*F* = 5.10, *p* = 0.0245) |

Note. BMI = body mass index; CG = control group; HIV = Human Immunodeficiency Virus; HTN = hypertension; IG = intervention group; OR = odd ratio; RCT = randomized controlled trial; RN = registered nurse; STI = Sexually transmitted infections; T2DM = type 2 diabetes mellitus.

References

Akintobi, T. H., et al. (2016) Outcomes of a behavioral intervention to increase condom use and reduce HIV risk among urban African American young adults. *Health Promotion Practice,* **17**, 751-719. doi: 10.1177/1524839916649367

Amirehsani, K. A., et al. (2019) Hispanic families' action plans for a healthier lifestyle for diabetes management. *Diabetes Educator,* **45**, 87-95. doi: 10.1177/0145721718812478

Briant, K. J., et al. (2018) Using a culturally tailored intervention to increase colorectal cancer knowledge and screening among Hispanics in a rural community. *Cancer Epidemiology, Biomarkers & Prevention,* **27**, 1283-1288. doi: 10.1158/1055-9965.EPI-17-1092

Desrosiers, A., et al. (2019) A randomized controlled pilot study of a culturally-tailored counseling intervention to increase uptake of HIV pre-exposure prophylaxis among young black men who have sex with men in Washington, DC. *AIDS and Behavior,* **23**, 105-115. doi: 10.1007/s10461-018-2264-5

DiClemente, R. J., Murray, C. C., Graham, T., & Still, J. (2015) Overcoming barriers to HPV vaccination: A randomized clinical trial of a culturally-tailored, media intervention among African American girls. *Human Vaccines & Immunotherapeutic,* **11**, 2883-2894. doi: 10.1080/21645515.2015.1070996

Falbe, J., et al. (2015) Active and healthy families: A randomized controlled trial of a culturally tailored obesity intervention for Latino children. *Academic Pediatrics,* **15**, 386-395. doi: 10.1016/j.acap.2015.02.004

Hall, M. A., Johnson-Turbes, A., Niles, P., & Cnatey-McDonard, S. (2011) Development and implementation of a culturally tailored, community-based intervention to raise awareness of brain health among African Americans. *Journal of Health Disparities Research and Practice,* **9**, 1-18

Im, E., et al. (2019) Decreasing menopausal symptoms of Asian American breast cancer survivors through a technology-based information and coaching/support program. *Menopause,* **26**, 373-382. doi: 10.1097/GME.0000000000001249

Larsen, B., et al. (2015) Cost effectiveness of a mail-delivered individually tailored physical activity intervention for Latinas vs. a mailed contact control. *International Journal of Behavioral Nutrition and Physical Activity,* **12**, 140. doi: 10.1186/s12966-015-0302-5

McEwen, M. M., Pasvogel, A., & Murdaugh, C. (2019) Effects of a family-based diabetes intervention on family social capital outcomes for Mexican American adults. *Diabetes Educator,* **45**, 272-286. https:// doi: 10.1177/0145721719837899

Okoro, F. O., Veri, S., & Davis, V. (2018) Culturally appropriate peer-led behavior support program for African Americans with type 2 diabetes. *Front Public Health,* **6***,* 340. doi: 10.3389/fpubh.2018.00340. eCollection 2018

Pinsker, E. A., et al. (2017) The development of culturally appropriate tobacco prevention videos targeted toward Somali youth. *Progress in Community Health Partnerships,* **11**, 129-136. doi: 10.1353/cpr.2017.0017.

Printz, C. (2019) Culturally tailored intervention increases HPV vaccination rates among Asian American adolescents. *Cancer,* **125**, 1583. doi: 10.1002/cncr.32155.

Ramirez, A G., et al. (2019) Improving quality of life among Latino cancer survivors: Design of a randomized trial of patient navigation. *Contemporary Clinical Trials,* **76**, 41-48. doi: 10.1016/j.cct.2018.11.002

Ravenell, J., et al. (2015) Tailored approaches to stroke health education (TASHE): Study protocol for a randomized controlled trial. *Trials,* **16***,* 176. doi: 10.1186/s13063-015-0703-4

Schoenthaler, A., et al. (2015) A practice-based randomized controlled trial to improve medication adherence among Latinos with hypertension: Study protocol for a randomized controlled trial. *Trials,* **16**, 290. doi: 10.1186/s13063-015-0815-x

Shi, Z., et al. (2018) Psychosocial mediators of dietary change among Hispanic/Latina breast cancer survivors in a culturally tailored dietary intervention. *Psychooncology,* **27**, 2220-2228. doi: 10.1002/pon.4799

Shrestha, S., et al. (2017) Calmer life: A hybrid effectiveness-implementation trial for late-life anxiety conducted in low-income, mental health-underserved communities. *Journal of Psychiatric Practice,* **23**, 180-190. doi: 10.1097/PRA.0000000000000234

Weber, M. B., et al. (2016) The stepwise approach to diabetes prevention: Results from the D-CLIP randomized controlled trial. *Diabetes Care,* **39**, 1760-1767. doi: 10.2337/dc16-1241

Yeh, M., et al. (2016) Translation of the diabetes prevention program for diabetes risk reduction in Chinese immigrants in New York city. *Diabetic Medicine,* **33**, 547-551. doi: 10.1111/dme.12848
